# Enhancing Executive Function Skills in Children With Attention-Deficit/Hyperactivity Disorder via Immersive Virtual Reality Interventions: Scoping Review

**DOI:** 10.2196/57225

**Published:** 2024-11-22

**Authors:** Asli Konaç, Maristella Bini, Naomi Fusco, Pierre Bourdin-Kreitz

**Affiliations:** 1 eHealth Department, Universitat Oberta de Catalunya, Barcelona, Spain; 2 Dipartimento di Psicologia, University of Campania “Luigi Vanvitelli”, Caserta, Italy; 3 Department of Computer Science, Multimedia and Telecommunication, Universitat Oberta de Catalunya, Rambla del Poblenou 156, Barcelona, 08018, Spain, 34 934505205; 4 XR-Lab of the Hub Interdisciplinari de Recerca i Innovació, Universitat Oberta de Catalunya, Barcelona, Spain

**Keywords:** immersive virtual reality, ADHD, neurofeedback, executive functions, systematic review, adolescent, attention-deficit/hyperactivity disorder, behavioral therapy, digital health tools, neurodiversity, virtual reality, digital mental health

## Abstract

**Background:**

This scoping review investigated immersive virtual reality (IVR) interventions for improving executive function skills of children and adolescents with attention-deficit/hyperactivity disorder (ADHD).

**Objective:**

This study aimed to identify and closely inspect the characteristics of these interventions and provide a summary of key findings to guide researchers in their future investigations.

**Methods:**

A search across Web of Science, Scopus, PubMed, and APA PsycInfo databases was carried out with restrictions of publication date (2000‐2023) and language (English). The inclusion criteria were (1) research articles, excluding protocols, book chapters, reviews, and meta-analyses; (2) usage of IVR, excluding computer-based VR or augmented reality technologies; (3) aim of targeting executive function skills; (4) sample of children and adolescents diagnosed with ADHD (with or without learning disorder comorbidity); and (5) intervention studies (quasi-experimental clinical trials and randomized controlled trials, excluding assessments). Finally, the characteristics of the studies were summarized and inspected.

**Results:**

The search yielded 2484 potential records. After a rigorous screening process, 6 articles (5 randomized controlled trials and 1 pilot study) were included. A certain heterogeneity in duration, designs of IVR interventions, and outcome measures were observed. All studies reported overall improvements in the attentional performances of children; however, only a few reported improvements in executive functions. In addition, a tendency toward integration of neurofeedback systems with IVR technologies was observed.

**Conclusions:**

Because of the specific objectives and related inclusion and exclusion criteria of this review, only a few interventions could be included and analyzed. Even though there seem to be promising applications of IVR for children and adolescents with ADHD, heterogeneity in intervention characteristics accompanied by observed overall high or serious risk of bias prevented the authors from making generalized conclusions.

## Introduction

Attention-deficit/hyperactivity disorder (ADHD) is one of the most common neurodevelopmental disorders in children and adolescents. Commonly diagnosed during childhood, the disorders often persist into adulthood [1]. Generally characterized by levels of inattention, impulsivity, and hyperactivity, ADHD leads to impairment in daily life [2]. To date, interventions for ADHD have included medication therapy, behavioral therapy (cognitive behavioral therapy [CBT]), or a combination of both [3]. While a patient undergoing CBT receives sessions involving cognitive and behavioral training, classical psychopharmacological treatment includes the prescription of methylphenidate dextroamphetamine or pemoline [4]. While these approaches proved effective for rehabilitation, they come with several drawbacks, such as time consumption, and in some cases, a lack of behavioral improvement [5,6]. Consequently, alternative options, particularly the use of technological tools, have been explored.

Recent technological advancements have led to the integration of interventions with the use of digital health tools. Digital health tools refer to the use of any kind of technology for the purpose of delivering health assessments and interventions. Such tools are in a wide range: from emails sent by doctors for informing patients to the use of virtual reality (VR) for treatment purposes [7,8]. For ADHD treatment, VR technologies have become one of the trending digital health tools [9,10].

VR can be defined as a sensory illusion achieved through computer technology consisting of hardware and software that simulates in real-time (physical) presence of users and their interaction in an environment that is virtually created. This definition is consistent with the literature [11] and independent of technology. Moreover, VR is considered a recent technology that started 50 years ago, and for more than 2 decades now, researchers and clinicians have been working with VR to build tools to enhance clinical research, assessment, and intervention [12]. The benefits of using this technology include increased immersion, feeling of presence, and motivation for patients during the treatment process which is determinant in the science of rehabilitation [12-15].

VR systems have been usually classified as either immersive or nonimmersive systems [16], and qualified as low- or high-immersive VR [17]. Low-immersive (or nonimmersive) VR refers to technological devices such as desktop computer screens, whereas high-immersive VR is primarily linked to head-mounted display (HMD) systems and Cave Automatic Virtual Environment systems [18]. There are criticisms about “desktop VR” in the literature because it does not allow the user to interact naturally in the virtual environment and fails to induce an immersive experience [14,16,18]. This is why, in this review, we focus on research settings where children can fully experience immersive virtual environments, therefore only including studies using immersive virtual reality (IVR).

The objective of this review is (1) to identify published IVR interventions that target executive function skills of children diagnosed with ADHD, (2) to closely inspect the characteristics of these interventions through descriptive or narrative analysis, and (3) to provide a summary of key findings to guide researchers in their future investigations.

## Methods

### Search Strategy

The articles included in this research have been collected from the search databases Web of Science, Scopus, PubMed, and APA PsycInfo. The initial search was carried out on October 11, 2022, with restrictions of publication date 2000‐2023 and English language by 2 independent reviewers (MB and NF) and lastly updated on December 29, 2023. The PICO (Patient, Population or Problem, Intervention, Comparison, and Outcome) model [19] was followed while deciding on the search terms (see Multimedia Appendix 1). The comparison and outcome part of the PICO model (see Multimedia Appendix 1) was not included among the search terms as it could restrict the search results [20].

All databases were searched with identical search terms. See Multimedia Appendix 2 for a detailed list of search terms for each database.

### Selection of Studies and Data Collection Process

After searching the databases, the data were extracted in Microsoft Excel format with the following information about the articles: DOI number, title of the articles, abstracts, authors, publication year, and name of the source title (search database). After exporting data from each database, it was combined into a single file and duplicates were subsequently eliminated. Two independent titles and abstract screening were carried out according to this information. Lastly, the full text of each article was examined by the 3 reviewers(AK, MB, and NF). Any disagreement between them over the eligibility of any study was resolved with an examination of the fourth reviewer (PB-K). The resulting studies were included in the final data according to the following criteria. (1) Only research articles were included; protocols, book chapters, reviews, and meta-analyses were excluded. (2) Only IVR techniques were included. (3) The target of the studies should be patients with ADHD, learning disorders, or both as comorbid diagnoses. (4) Only studies targeting children and adolescents were included. (5) Only intervention studies (quasi-experimental clinical trials and randomized controlled trials [RCTs]) were included.

A descriptive or narrative data analysis method was followed. The following information about the articles was extracted: country of the research, participant characteristics (sample size, diagnosis, and age), measures that are used in pre- and postassessments, and outcomes. A summary of this information is presented in Table 1.

**Table 1.** Summary of the characteristics of papers included in the review.

Reference	Study characteristics	Participant characteristics	Intervention groups	Measures (pre- and postassessment)	Outcome and results
Cho et al [21]	Country: Korea Design: RCT^a^	Number of participants: 50 Diagnosed with ADHD^b^: no Comorbidity: no Age: 14‐18 years	1. No intervention (control group) 2. Desktop VR^c^ electroencephalography biofeedback training 3. Desktop VR cognitive training 4. VR electroencephalography biofeedback training 5. VR cognitive training	For ADHD symptoms: CPT^d^	For ADHD symptoms: significant improvement for experimental groups (*P*<.01)
Cho et al [22]	Country: Korea Design: RCT	Number of participants: 28 Diagnosed with ADHD: no Comorbidity: no Age: 14‐18 years	1. No intervention (control group) 2. Non-VR group 3. VR group	For ADHD symptoms: CPT	For ADHD symptoms: statistically significant improvement in attention enhancement for the VR group (*P*<.01)
Bioulac et al [23]	Country: France Design: RCT	Number of participants: 51 Diagnosed with ADHD: yes Comorbidity: no Age: 14‐18 years	1. Placebo psychotherapy group 2. Methylphenidate group 3. Therapy by virtual remediation or VR group	For attention: a virtual classroom task For ADHD symptoms: ADHD-RS^e^ and CPT II	For attention: significantly higher scores for the VR group compared to the psychotherapy group (*P*<.0001) and the methylphenidate group (*P*<.0001)
Ou et al [24]	Country: Taiwan Design: Pilot	Number of participants: 3 Diagnosed with ADHD: yes Comorbidity: no Age: 8‐12 years	Three different immersive virtual reality games with HTC VIVE (HTC Corp) focusing on hand-eye and hand-foot coordination: Fishing Master Fruit Train Ocean Manager	For nonverbal intelligence: TONI-4^f^ For executive functions: WCST^g^ For attention: ATESC^h^ For ADHD and oppositional defiant disorder symptoms: SNAP-IV^i^	For nonverbal intelligence: overall improvement For executive functions: overall improvement For attention: overall improvement For ADHD and oppositional defiant disorder symptoms: overall improvement
Skalski et al [25]	Country: Poland Design: RCT	Number of participants: 87 Diagnosed with ADHD: yes Comorbidity: no Age: 9‐15 years	1. Standard HEG BFB^j^ group (desktop) 2. VR HEG BFB^k^ with distractors (2D) 3. VR HEG BFB without distractors (3D)	For attention: The visual search task The multitasking test The short form of Mackworth Clock Task	For attention: better performance of children in VR HEG BFB groups (*P*<.0001)
Schena et al [26]	Country: Italy Design: RCT	Number of participants: 60 Diagnosed with ADHD: yes Comorbidity: learning disorder Age: 5‐12 years	1. Traditional (conventional) therapy 2. VR therapy with the IAmHero system	For attention and concentration skills: BIA^l^ For ADHD symptoms and behavioral disorders: Conners-3 questionnaire For executive functions: TOL^m^	For attention and concentration skills: improvement in areas of attentional processes and sustained auditory attention (*P*<.05) For ADHD symptoms and behavioral disorders: improvement especially for hyperactivity or impulsivity subtype (*P*<.05) For executive functions: improvement in task planning and organization (*P*<.05)

a RCT: randomized controlled trial.

b ADHD: attention-deficit/hyperactivity disorder.

c VR: virtual reality.

d CPT: continuous performance test.

e ADHD-RS: attention-deficit/hyperactivity disorder rating scale.

f TONI-4: Test of Nonverbal Intelligence, Fourth Edition.

g WCST: Wisconsin Card Sorting Test.

h ATESC: attention test for elementary school children.

i SNAP-IV: Swanson, Nolan, and Pelham Questionnaire Version 4.

j HEG BFB: hemoencephalographic biofeedback.

k VR HEG BFB: hemoencephalographic biofeedback with virtual reality.

l BIA: Italian Battery of ADHD.

m TOL: Tower of London test.

The common and different points of the articles on their outcomes, assessments, and intervention characteristics are explained and summarized in the Results section.

## Results

### Study Selection and Characteristics

**Figure 1.**
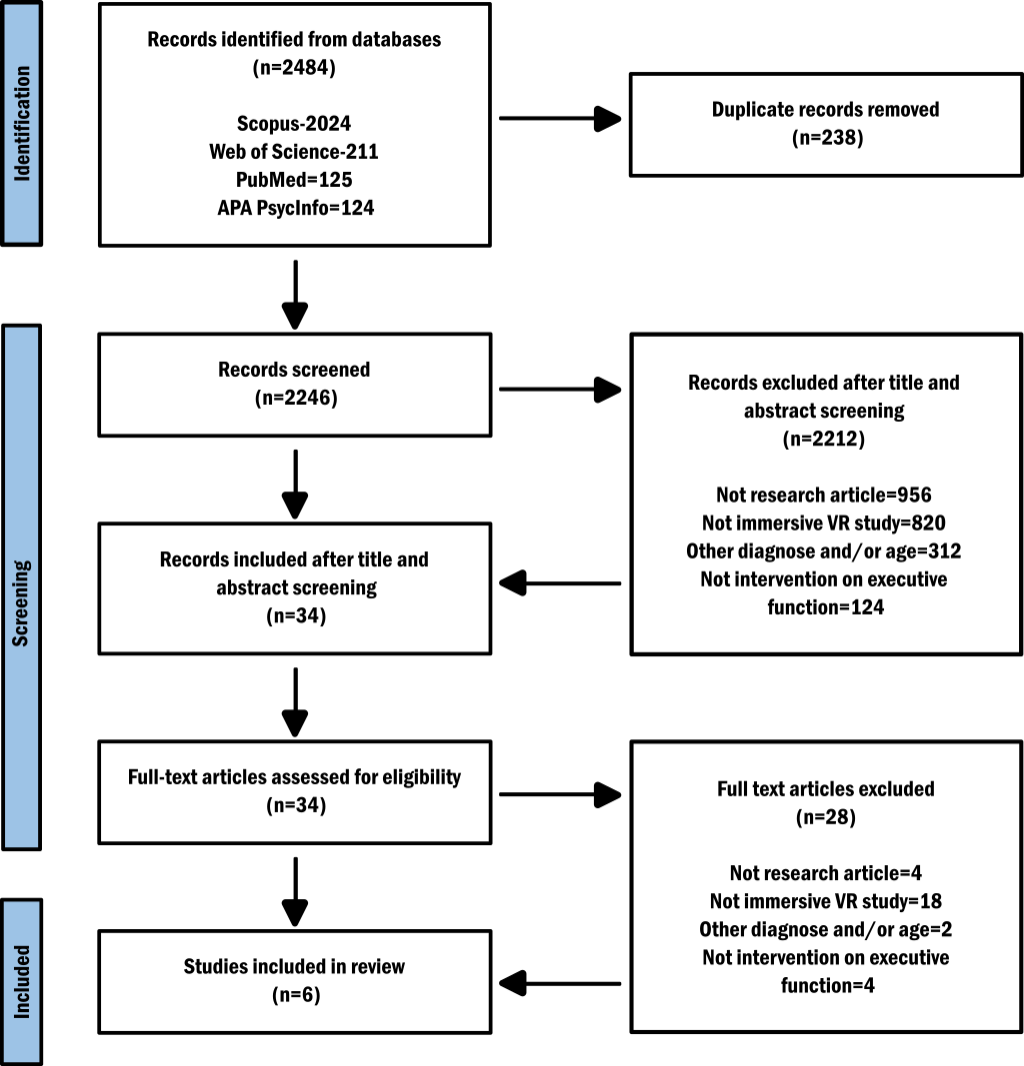
PRISMA (Preferred Reporting Items for Systematic Reviews and Meta-Analyses) flow diagram.

The PRISMA (Preferred Reporting Items for Systematic Reviews and Meta-Analyses) flow diagram is shown in Figure 1. Initially, 2484 articles were identified through 4 databases; after removing the duplicates, the remaining 2246 were screened based on the titles and abstracts. The results of this screening process were the identification of 34 studies, and the exclusion of 2212 studies, based on the inclusion and exclusion criteria. Three independent reviewers (AK, MB, and NF) assessed the methodological quality of the studies included. In the first screening phase, the criteria that led to the greatest exclusion of articles were criterion 1 (not research article) and criterion 2 (not immersive VR study). Precisely 956 articles were excluded for criterion 1, and 820 for criterion 2.

Later, the 34 identified articles were downloaded for a full-text screening. Of these, 28 were excluded for various reasons: being book chapter or protocol (4), using non-immersive technologies (18), study samples within other diagnoses and age groups (2), not being intervention but assessment on executive functions (4). Finally, 6 articles were included in the review.

### Evaluation Outcomes

#### Origin of Studies

The selected articles originate from Asia (3 articles) and Europe (3 articles). There are no articles from the United States.

#### Study Design

The review included 5 RCTs [21-23,25,26] and 1 pilot study [24]. The pilot study was included in the review because of its design characteristics, which is explained below. Except for the pilot study, all 5 studies included control and comparison groups in their experimental designs.

#### Participant Characteristics

Among the studies, there were variations in the ages of the participants. Cho et al [21] included 50 and Cho et al [22] included 28 children with the oldest age range (14-18 years), while Bioulac et al [23] included 51 children aged between 7 and 11 years, and Schena et al [26] included 60 children aged between 5 and 12 years. The pilot study of Ou et al [24] included 3 children in their sample who were aged between 8 and 12 years. Skalski et al [25] included 90 children, the widest participant’s age range (9-15 years).

For diagnoses of children, Ou et al [24] did not provide clear information about the diagnoses of children, as they stated “had some difficulty in learning in school, and they were inattentive, impulsive, hyperactive, and distracted. Although they were not officially diagnosed as ADHD, about 30% of them most likely had ADHD.” Cho et al [21,22] also mentioned their participants as “not officially having diagnoses of ADHD, but they had some difficulty in learning and were inattentive, impulsive, hyperactive and distracted.” The remaining 3 studies stated children’s diagnoses as ADHD. Among 6 articles, only Schena et al [26] did not exclude comorbidity of learning disorders in their sample.

#### Characteristics of VR Interventions

##### Overview

Although there are variations in VR technology, primarily resulting from the 20-year gap between the oldest and most recent publications, all the studies included in this review used immersive VR interventions. Thus, they provide an interactive and engaging environment for the assessment or treatment of ADHD symptoms and executive dysfunctions in children. However, there are variations in intervention designs and duration across the publications.

##### Design of Interventions

Cho et al [21] presented an electroencephalography biofeedback system combined with IVR specifically designed for treating ADHD in a virtual classroom, which is called virtual reality cognitive training; the authors compare this training between groups either with VR, or only with a computer screen and no intervention group. Cho et al [22] compared HMD usage for neurofeedback training in a virtual classroom with its only computer screen usage. Skalski et al [25] used hemoencephalography biofeedback and compared its use in IVR, desktop VR, and the standard version (2D game presented on a 21-inch television screen). Bioulac et al [23] compared virtual remediation with methylphenidate, as the only study in this review that compared the effectiveness of medical intervention with an IVR design.

Two studies [24,26] designed and conducted game-like interventions within IVR. Ou et al [24] focused on VR rehabilitation games for ADHD symptoms and developed three different games for their pilot study: (1) Fishing Master, for improving hand-eye coordination; (2) Fruit Train, for improving physical coordination of upper and lower limbs; and (3) Ocean Manager, for improving visuomotor skills. They tested these games without involving comparison/control groups. Schena et al [26] used the IAmHero tool, which targets ADHD symptoms and executive dysfunctions. It consists of three games: (1) Topological Categories, for improving visual-spatial orientation, motor coordination, planning, and selective auditory attention; (2) Infinite Runner, for improving visuomotor skills; and (3) Space Coding, for improving motor skills, planning, visuospatial and constructive skills, reasoning, and problem-solving.

##### Duration of Interventions

The duration of the interventions lasted from 1.5 to 6 months, with the frequency ranging between 1 and 3 times per week. In the studies of Cho et al [21,22], subjects in both experimental and placebo groups underwent 8 sessions over 2 weeks, each session lasting for 20 minutes. The control group did not receive any training sessions in the meantime. These sessions were conducted twice a week for 6 weeks. In the study of Ou et al [24], the participants went through 36 sessions. These sessions were conducted 3 times a week for 3 weeks within a 3-week cycle for each game. Each session was divided into three 10-minute segments. In the study of Skalski et al [25], the experimental group underwent 10 sessions. These sessions were conducted once a week, and each session was divided into three 10-minute segments. In the study of Schena et al [26], the trials lasted for 6 months; the experimental group received weekly 30 minutes of IVR intervention within each 50 minutes (the remaining 20 minutes were designed as free play in the therapy room) as an addition to their ongoing therapy sessions from the center that they receive treatment. The control group received weekly sessions (2 per week) of speech and psychomotor treatment, which are designed based on the patient’s clinical needs.

### Assessments and Outcomes

#### Assessments

The authors observed heterogeneity in the selection of assessments and outcome variables.

Cho et al [21] conducted continuous performance test (CPT) assessments before and after the training sessions to measure attention and impulsivity in all the participants. CPT scores were analyzed and compared between the 3 groups: experimental group 1 (VR), experimental group 2 (non-VR group), and no-intervention control group. Cho et al [22] conducted CPT assessments before and after the training sessions to measure attention and impulsivity in all the participants. CPT scores were analyzed and compared between the 5 groups: experimental group 1 (virtual reality electroencephalography biofeedback training), experimental group 2 (VR cognitive training), placebo group 1 (desktop VR electroencephalography biofeedback training), placebo group 2 (desktop VR cognitive training), and control group (no intervention).

Bioulac et al [23] conducted pre- and postassessments for participants’ performance in (1) attention with the visual classroom task and (2) ADHD symptoms with the ADHD rating scale (ADHD-RS) and continuous performance test II (CPT II).

The pilot study of Ou et al [24] was conducted before and after assessments for participants’ ADHD symptoms with CPT II for each experimental session. The authors considered several variables, such as response time, SD of the response time (as a measure of variability or consistency), variability, errors of commission (as a measure of impulsivity or failure to inhabit response), errors of omission (as a measure of inattention), and response sensitivity (as a measure of diminishing performance over time).

In the study of Skalski et al [25], the participants were assessed for their intelligence at the recruitment stage with Raven’s colored progressive matrices in Polish standardization. For the interventions, the participants were assessed for their selected aspects of attention with (1) the short form of the Mackworth Clock Task for vigilance, (2) the visual search task for attention in the conjunctive search paradigm, and (3) the multitasking test for divided attention (see Multimedia Appendix 3 for details of assessment procedures).

In the study of Schena et al [26], participants were assessed for their cognitive profile in the recruitment stage with the Wechsler Intelligence Scale for Children, 4th edition. In the pre- and postintervention phases, participants were assessed with the Italian Battery of ADHD for their attention and concentration skills*,* with the Conners-3 questionnaire for the assessment of ADHD and behavioral disorders, and finally with the Tower of London test for assessing their strategic decision-making and problem-solving skills.

#### Outcomes

All the studies reported improvements in the attentional performances of children by looking at differences between the pre- and postassessments. The pilot study of Ou et al [24] provided only a comparison between numerical values without any statistical analyses. The studies of Cho et al [21,22], Bioulac et al [23], Skalski et al [25], and Schena et al [26] presented their results with statistically significant results.

The analysis of Cho et al [21] showed that there were significant improvements for experimental groups in the number of correct answers, omission errors, and response sensitivity (*P*<.01). The control group indicated no significant change. There was a reduction in commission errors and response times for experimental groups too, but those results were not statistically significant.

The analysis of Cho et al [22] showed that the main effect for the measurement time (*F*_1,25_=39.775, *P*<.01) and the interaction effect of group × the measurement time (*F*_2,25_=8.715, *P*<.01) were significant, thus the number of hits in CPT II of the VR group increased compared to the non-VR and control groups. Regarding the reaction time in CPT II, the analysis showed a significant main effect for the measurement time (*F*_1,25_=8.545, *P*<.01) indicating a decrease in the reaction time of the VR group after training, implying that participants paid more attention to the tasks. Additionally, for the omission error in CPT, the main effect for measurement time (*F*_1,25_=31.179, *P*<.01) and the interaction effect of a group × the measurement time (*F*_2,25_=7.273, *P*<.01) were significant. Omission errors for the VR group decreased further than for non-VR and control groups. After training, both groups showed fewer commission errors. The main effect of the measurement time on commission errors (*F*_1,25_=5.698, *P*<.05) and response bias (*F*_1,25_=7.724, *P*<.01) were significant.

The analysis of Bioulac et al [23] showed that for virtual classroom task, there were significant differences in the number of correct hits for all the groups (*F*_2,47_=14.56, *P*<.0001) and for the number of commissions (*F*_2,47_=3.01, *P*=.05). The number of correct hits for the VR group was significantly higher than the psychotherapy group (*P*<.0001) and the methylphenidate group (*P*<.0001). For the number of commissions, the VR group was significantly lower than the methylphenidate group (*P*<.0001), while it was equivalent to methylphenidate and psychotherapy groups. For ADHD-RS, there were significant differences between the groups on ADHD-RS total (*F*_2,45_=20.98, *P*<.0001).

The pilot study of Ou et al [24] reported an overall improvement in nonverbal intelligence, executive functions, attention and symptoms of ADHD, and oppositional defiant disorder.

The analyses of Skalski et al [25] showed that children who participated in hemoencephalographic biofeedback with virtual reality (VR HEG BFB) with distractors (2D) and VR HEG BFB without distractors (3D) performed significantly better than children who participated in the standard HEG BFB (desktop) group with regard to all dependent variables of the measurement: omission errors (Group A: *P*=.018; Group B: *P*=.002), commission errors (Group A: *P*=.007; Group B: *P*=.003), response time slope in visual search (Group A: *P*=.007; Group B: *P*<.001), single tasks (Group A: *P*=.011; Group B: *P*=.006), as well as multitasks (Group A: *P*=.021; Group B: *P*<.001).

The analyses of Schena et al [26] showed that there is a significant improvement in participants’ mean scores between pre-test (*t*_0_) and post-test (*t*_1_) assessments in areas of attentional processes, problem-solving (mean*_t_*_0_ 94, SD 12.76; mean*_t_*_1_ 97.52, SD 5.47; *P*<.05) sustained auditory attention (mean*_t_*_0_ 7.21, SD 0.65; mean*_t_*_1_ 9, SD 0.36*; P<*.05) and executive functions (ie, task planning and organization; mean*_t_*_0_ 24.45, SD 5.65; mean*_t_*_1_ 27.79, SD 3.66, *P<*.05).

## Discussion

### Principal Results

The main goal of this scoping review was (1) to identify published IVR interventions that target executive function skills of children diagnosed with ADHD, (2) to closely inspect the characteristics of these interventions through descriptive or narrative analysis, and (3) to provide a summary of key finding and produce recommendations to guide researchers for their future investigations. Identify and analyze the characteristics of IVR interventions for improving executive function skills of children with ADHD.

The first point catching attention is the very limited number of publications that met the selection criteria. Indeed, our results show that among more than 2000 articles published in the last 22 years, only 6 studies meet our inclusion criteria. These criteria encompass research articles from 2000 to 2023 considering IVR interventions for children diagnosed with ADHD, learning disorders, or both as comorbid conditions. When looking more in detail, the 2 criteria which eliminated the greatest number of publications are “not research article,” with 956 articles discarded, and “not immersive VR” with 820 articles discarded. This shows that almost half of the articles we found were not research articles. That is to say that during the last 22 years, a large part of the published articles concerning ADHD were not experimental studies but generic articles, protocols, book chapters, reviews, and meta-analyses. This is intriguing, as it suggests that despite ADHD being a significant and actively researched subject, nearly half of the scientific literature on this topic does not directly report experiments, or only partially. This might indicate that experimental studies on ADHD present considerable challenges or at least that experimentally studying ADHD is more difficult than it seems especially when using IVR.

The second most discriminating exclusion criterion, the use of IVR, can probably be explained by the fact that initially IVR was not easily accessible and reserved for laboratories with large financial resources or specialized in IVR. Hence, the preference is for a less immersive setup that is more technologically and economically accessible. One can guess that with the democratization of IVR, there will be an augmentation of the IVR experimental setup. Indeed, with the falling cost of VR technologies and the development of high-quality HMDs, the use of IVR in learning environments may become more widespread in the future [27-29].

On the other hand, our findings revealed heterogeneity in the study designs (5 RCTs and 1 pilot study). Furthermore, there were variations in the outcome measures, except for the Continuous Performance Test (editions I and II), which was commonly preferred by Cho et al [21,22] and Bioulac et al [23]. All studies involved children and adolescents diagnosed with ADHD or exhibiting possible symptoms observed by the researchers. The authors included learning disorder in the database searches, considering the high comorbidity rate between ADHD and learning disorder. However, only 1 of the 6 studies [26] included participants diagnosed with learning disorder as comorbid. Regarding VR interventions, there was a tendency to combine biofeedback (ie, neurofeedback) systems with IVR technologies.

As highlighted by this scoping review, the use of biofeedback (ie, neurofeedback) systems appears to be a trending digital health tool for the treatment of developmental disorders, particularly ADHD [30-32]. The objective of these interventions was to train and reinforce patients for desired cortical activities, which were observed with electroencephalography signals. Indeed, cortical activities of patients with ADHD were found to differ in comparison with healthy individuals for the desired tasks [31,32]. The interest of researchers in using biofeedback systems for ADHD stems from “side effects and inadequate response to current medical treatments” [31], but also because certain patients with ADHD did not benefit from CBT interventions [32]. With the application through integrations of biofeedback with VR systems, we can expect to see more interventions in the future that combine biofeedback with IVR.

### Comparison With Prior Work

Previous reviews with similar objectives also found a small sample size. For instance, a recent study [9] aimed to evaluate the effectiveness of VR-based interventions (without specifying a target cognitive skill) for children with ADHD and could only include 6 studies for its qualitative analysis and 4 for quantitative analysis. Another study published the same year as [33] and looking at VR interventions with children (without specifying any mental health disorder) only included 19 studies. These findings, combined with our results, highlight the scarcity of RCTs investigating the use of IVR specifically for children.

Furthermore, the literature highlighted some aspects that indicate the potential benefits of this technology for children. First, IVR could significantly increase the ecological validity and reliability of behavioral interventions. In other words, the abilities that were gained by interventions might be more easily transformed to the daily settings of children, since the tool itself gave the opportunity to create environments that are similar to real-world environments [34].

Second, children might become more motivated to the rehabilitation process by the use of up-to-date technologies (ie, VR) since it can make the process more enjoyable for them. Previous work in the literature indicated that treatments that were more enjoyable for children and adults could lead the treatment process to be easier, healthier, and more effective [14,35,36]. As mentioned before, this review included interventions providing interactive and engaging environments for children, which refers to “game-like” applications.

In further research with IVR, game design elements can be provided with a greater sense of interactivity owing to its immersive experience, which can increase user engagement and benefits of interventions [32].

### Limitations

This scoping review has certain limitations. First, due to the specific objectives and related inclusion and exclusion criteria of the study, only a few interventions could be included and analyzed.

Another limitation of this study is the use of the PICO framework while deciding on search terms. However, built-in features of databases such as MeSH (Medical Subject Heading) terms were not used, which could limit the results.

### Conclusions

Our study showed that only 6 studies were in line with our inclusion criteria. A certain heterogeneity in the study designs and outcome measures was observed.

All the studies included participants with diagnoses (or possible symptoms observed by researchers) of ADHD and only 1 of the 6 studies included participants with learning disorders as comorbidity. The age range of the participants varied between the studies.

For IVR interventions, a tendency to combine biofeedback systems with immersive reality technologies was observed. However, this observation cannot be generalized due to the limited sample size. To test the advantages of IVR and the effectiveness of the interventions with systematic reviews and meta-analyses, more research should be conducted. As Ou et al [24] suggested, further research should also investigate suitable designs for different age and gender groups.

About 60 years ago, Ivan Sutherland developed the first HMD system called the “Sword of Damocles” [37]. However, it was only about 15 years ago that significant advancements were made in both hardware and software, permitting the rise of consumer VR headsets and the democratization of IVR. Hence, it seems clear that IVR is a promising technology with encouraging results and great potential for many applications. However, it also seems clear that this is a relatively young technology that requires more comprehensive and more in-depth studies, in particular.

## Supplementary material

10.2196/57225 Multimedia Appendix 1 PICO (Patient problem, Intervention, Comparison, and Outcome) model.

10.2196/57225 Multimedia Appendix 2 Search strategies.

10.2196/57225 Multimedia Appendix 3 Assessment procedures.

10.2196/57225 Checklist 1 PRISMA-ScR (Preferred Reporting Items for Systematic reviews and Meta-Analyses extension for Scoping Reviews) checklist.

## Abbreviations

ADHD: attention-deficit/hyperactivity disorder
ADHD-RS: attention-deficit/hyperactivity disorder rating scale
CBT: cognitive behavioral therapy
CPT: continuous performance test
CPT II: continuous performance test II
HMD: head-mounted display
IVR: immersive virtual reality
MeSH: Medical Subject Heading
PICO: Patient problem, Intervention, Comparison, and Outcome
PRISMA: Preferred Reporting Items for Systematic Reviews and Meta-Analyses
RCT: randomized controlled trial
VR: virtual reality
VR HEG BFB: hemoencephalographic biofeedback with virtual reality
