# Supplementary material for: Enhancing Executive Function Skills in Children With Attention-Deficit/Hyperactivity Disorder via Immersive Virtual Reality Interventions: Scoping Review
Source: JMIR XR Spat Comput. 2024 Nov 22;1:e57225. doi: 10.2196/57225 (PMC13202507; doi:10.2196/57225)
Supplement: Multimedia Appendix 1 [file xr-v1-e57225-s001.docx]

**PICO Model**

The PICO model [1] was followed by the researchers to define search terms, research questions and eligibility criteria. The PICO model includes four parts: patients or population (P), intervention (I), comparison (C), and outcome (O). Articles were selected in accordance with the PICO guidelines as follows:

P) Children (age< 18) with attention deficit hyperactivity disorder

I) Immersive- Virtual Reality based treatment or rehabilitation

C) Computer based or classical paper-pencil interventions or no intervention control groups

O) Improvement in executive functions (including attention and working memory).

1. Schardt, C., Adams, M. B., Owens, T., Keitz, S., & Fontelo, P. (2007). Utilization of the PICO framework to improve searching PubMed for clinical questions. *BMC Medical Informatics and Decision Making*, 7, 16. doi:<http://dx.doi.org/10.1186/1472-6947-7-1>

This is a Multimedia Appendix to a full manuscript published in the JMIR XR Spatial Computing. For full copyright and citation information see http://dx.doi.org/10.2196/jmir.57225
