# Supplementary material for: Enhancing Executive Function Skills in Children With Attention-Deficit/Hyperactivity Disorder via Immersive Virtual Reality Interventions: Scoping Review
Source: JMIR XR Spat Comput. 2024 Nov 22;1:e57225. doi: 10.2196/57225 (PMC13202507; doi:10.2196/57225)
Supplement: Multimedia Appendix 2 [file xr-v1-e57225-s002.docx]

**Search Strategies**

**PubMed**

(((((Virtual Reality) OR (VR)) AND (((Executive Function*) OR (Working Memory)) OR  (Attention)))  AND (((Rehabilitation) OR (Treatment)) OR (Intervention))) AND ((((((((ADHD) OR (ADD)) OR (Attention Deficit Hyperactivity Disorder)) OR (Attention Problem)) OR (Dyslex*)) OR (Learning Disorder)) OR (Reading Disorder)) OR (Reading Disability))) AND ((Child*) OR (Adolescen*))

Date restriction: 2000-2023

Language: English

Population: Humans

Results: **125**

**Web Of Science**

ALL=((Virtual Reality OR VR) AND (Executive Function* OR Working Memory OR Attention) AND (Rehabilitation OR Treatment OR Intervention) AND (ADHD OR Attention Deficit Hyperactivity Disorder OR Attention Problem OR Dyslex* OR Learning Disorder OR Reading Disorder OR Reading Disability) AND (Child* OR Adolescen*))

Date restriction: 2000.01.01 -2023-12-29

Language: English

Results: **221**

**APA PsycINFO**

(Virtual Reality OR VR) AND (Executive Function* OR Working Memory OR Attention) AND (Rehabilitation OR Treatment OR Intervention) AND (ADHD OR Attention Deficit Hyperactivity Disorder OR Attention Problem OR Dyslex* OR Learning Disorder OR Reading Disorder OR Reading Disability) AND (Child* OR Adolescen*)

Date restriction: 2000-2023

Language: English

Population: Humans

Results: **124**

**Scopus**

("Virtual Reality" OR "VR" ) AND ( "Executive Function*" OR "Working Memory" OR "Attention" ) AND ( "Rehabilitation" OR "Treatment" OR "Intervention" ) AND ( "ADHD" OR "Attention Deficit Hyperactivity Disorder" OR "Attention Problem" OR "Dyslex*" OR "Learning Disorder" OR "Reading Disability" OR "Reading Disorder" ) AND ( "Child*" OR "Adolescen*" )

Date restriction: 2000-2023

Language: English

Population: Humans

Results: **2024**

--- initial search (11.10.2022) ---

**Scopus**

( "Virtual Reality" OR "VR" ) AND ( "Executive Function*" OR "Working Memory" OR "Attention" ) AND ( "Rehabilitation" OR "Treatment" OR "Intervention" ) AND ( "ADHD" OR "Attention Deficit Hyperactivity Disorder" OR "Attention Problem" OR "Dyslex*" OR "Learning Disorder" OR "Reading Disability" OR "Reading Disorder" ) AND ( "Child*" OR "Adolescen*" )

Filters: Population (Human), Language (English), publication date (2000-2022)

Results: **1595**

**Medline**

("Virtual Reality" OR "VR") AND ("Executive Function*" OR "Working Memory" OR "Attention") AND ("Rehabilitation" OR "Treatment" OR "Intervention") AND ("ADHD" OR "Attention Deficit Hyperactivity Disorder" OR "Attention Problem" OR "Dyslex*" OR "Learning Disorder" OR "Reading Disability" OR "Reading Disorder") AND (Child* OR Adolescen*)

Filters: Human, English, publication date: 2000-2022

Results: **92**

**Web Of Science**

ALL=(Virtual Reality ) OR ALL=(VR) AND ((ALL=(Executive Function*)) OR ALL=(Working Memory )) OR ALL=(Attention) AND ((ALL=(Rehabilitation)) OR ALL=(Treatment)) OR ALL=(Intervention) AND ((((((ALL=(ADHD)) OR ALL=(Attention Deficit Hyperactivity Disorder)) OR ALL=(Attention Problem)) OR ALL=(Dyslex*)) OR ALL=(Learning Disorder)) OR ALL=(Reading Disorder)) OR ALL=(Reading Disability)AND (ALL=(Child*)) OR ALL=(Adolescent*)

Filters: Language (English), Publication Date (2000-2022)

Results: **190**

**APA PsycINFO**

(Virtual Reality OR VR) AND (Executive Function* OR Working Memory OR Attention) AND (Rehabilitation OR Treatment OR Intervention) AND (ADHD OR Attention Deficit Hyperactivity Disorder OR Attention Problem OR Dyslex* OR Learning Disorder OR Reading Disorder OR Reading Disability) AND (Child* OR Adolescen*)

Filters: Language (English), Publication Date (2000-2022), Population (Human)

Results: **110**

**PubMed**

(((((Virtual Reality) OR (VR)) AND (((Executive Function*) OR (Working Memory)) OR (Attention))) AND (((Rehabilitation) OR (Treatment)) OR (Intervention))) AND ((((((((ADHD) OR (ADD)) OR (Attention Deficit Hyperactivity Disorder)) OR (Attention Problem)) OR (Dyslex*)) OR (Learning Disorder)) OR (Reading Disorder)) OR (Reading Disability))) AND ((Child*) OR (Adolescen*))

Filters: Population (Human), Language (English), publication date (2000-2022)

Results: **74**

This is a Multimedia Appendix to a full manuscript published in the JMIR XR Spatial Computing. For full copyright and citation information see http://dx.doi.org/10.2196/jmir.57225
