# Supplementary material for: Enhancing Executive Function Skills in Children With Attention-Deficit/Hyperactivity Disorder via Immersive Virtual Reality Interventions: Scoping Review
Source: JMIR XR Spat Comput. 2024 Nov 22;1:e57225. doi: 10.2196/57225 (PMC13202507; doi:10.2196/57225)
Supplement: Multimedia Appendix 3 [file xr-v1-e57225-s003.docx]

**Details of Assessment Procedures of Skalski et al. (2021) [25]**

The Short Form of the Mackworth Clock Task was used to measure vigilance. In this test the subject's task is to monitor the hand of a clock on a screen. The hand moves in regular jumps every 1 second, but at rare and irregular intervals it makes a 2-second jump. The probability of irregular jumps is 6%, that is, out of 300 jumps only 18 are irregular. The subject must, therefore, identify the irregular jumps of the clock hand. Researchers record both the number of omissions and commission errors.

The Visual Search Task was used to assess attention in the conjunctive search paradigm in which the subject has to identify the red T letter among upside-down red T letters and blue T letters. The probability of occurrence of red T letters is 40 %. The probability of appearance of the red T letter is 40 %. This means that out of 48 boards with 520 items displayed, the red T letter appears in 19 boards. Researchers record the mean reaction time (RT) slope.

The Multitasking Test was used to assess divided attention. In this test a screen is divided in two parts, the subject reacts according to the position of the stimulus: In the upper part of the screen, the individuals react to the shape of the figure (with a square or rhombus), while in the lower part, they react to the number of dots inside those figures (two or three dots). The test is composed of 24 boards for single tasks, one stimulus feature: figure shape or number of dots inside the figure and 24 for multi-tasks, two stimulus features: figure shape and number of dots. For correct answers was recorded the mean reaction time in both single and multitasks.

This is a Multimedia Appendix to a full manuscript published in the JMIR XR Spatial Computing. For full copyright and citation information see http://dx.doi.org/10.2196/jmir.57225
